# PReS-FINAL-2010: Predictive value of whole-body MRI in juvenile dermatomyositis

**DOI:** 10.1186/1546-0096-11-S2-P23

**Published:** 2013-12-05

**Authors:** C Malattia, A Madeo, M Dellepiane, A Pistorio, MB Damasio, S Viola, S Pederzoli, A Beltramo, D Beleva, A Consolaro, A Martini

**Affiliations:** 1 Pediatrics, Istituto G. Gaslini, Genova, Italy; 2 Radiology, Istituto G. Gaslini, Genova, Italy

## Introduction

One of the major challenges in the management of juvenile dermatomyositis (JDM) is to accurately assess disease activity in order to optimize therapeutic strategies. Whole-body(WB)-MRI enables a reliable analysis of the site (skeletal muscle, subcutaneous tissue, myofascia) and magnitude of inflammatory process throughout the entire body. By providing an accurate estimation of the total disease activity load, this technique could be useful to tailor treatment according to disease severity.

## Objectives

To explore the potential value of WB-MRI in predicting treatment efficacy in JDM.

## Methods

WB-MRI was performed on a 1.5 T MRI scanner (using STIR sequences) to all consecutive JDM patients with disease duration ≤ 2 months, seen at the study Department between March 2010 and February 2013. Muscle signal abnormalities and subcutaneous tissue/myofascial involvement were assessed using a recently validated WB-MRI scoring system. Treatment efficacy was assessed at 3-months follow-up visit using the PRINTO criteria for the evaluation of response to therapy in JDM.

## Results

21 patients (9 boys, 12 girls, median age 6,6 years) were included. Four patients were treated with prednisone alone, while the others received prednisone in different combinations with methotrexate (N = 15) or cyclosporine (N = 2). Eleven patients (52.4%) met the PRINTO criteria for improvement at 3-months follow-up visit. WB-MRI muscular score was significantly higher in non-responders (median value: 61.2; IQR 53.5-65.5) compared to the responders (34.5; IQR 18-55 p = 0.001). A WB-MRI muscle score >57 was predictive of a poor response to treatment, as evaluated by ROC curve analysis (AUC:0,9; IC95%: 0,70-0,99; sensitivity: 100%, specificity: 70% + LR: 3,33 - LR: 0,00). Non-responders showed also a significant higher myofascial score (median value 1.5; IQR 0-3.5) compared to the improved patients (0; IQR 0-1.5 p = 0.04); no significant difference in subcutaneous involvement was found between responders (0.5; IQR 0-1.5) and non-responders (2.5; IQR 0.5-7; p = 0.08). Seven out of 8 patients (87.5%) with diffuse and homogeneous pattern of distribution of muscle inflammation were not improved at 3 months follow-up visit; *viceversa *10 out of 13 patients with the typical patchy distribution of muscle signal abnormalities were improved (p = 0.02).

## Conclusion

High WB-MRI muscular and myofascial scores and the diffuse and homogeneous pattern of muscle inflammation were associated with a more severe disease course. WB-MRI represents a promising tool to identify patients who could benefit from a more aggressive therapeutic regimen since the early stages of the disease.

## Disclosure of interest

None declared.

